# Musculoskeletal pain and health-related quality of life in Spanish health sciences university students

**DOI:** 10.23938/ASSN.1115

**Published:** 2025-10-03

**Authors:** Beatriz Rodríguez-Romero, Lucía López-López, Inés Fernández-Fraga, Sonia Pértega-Díaz

**Affiliations:** 1 Universidade da Coruña Psychosocial Intervention and Functional Rehabilitation Research Group Department of Physiotherapy, Medicine and Biomedical Sciences Oza A Coruña Spain; 2 Universidade da Coruña Faculty of Physiotherapy Oza A Coruña Spain; 3 Universidade da Coruña University School of Nursing Oza A Coruña Spain; 4 Instituto de Investigación Biomédica de A Coruña (INIBIC) Nursing and Health Care Research Group Xubias de Arriba A Coruña Spain; 5 Universidade da Coruña Rheumatology and Health Research Group Department of Health Sciences, Faculty of Nursing and Podiatry Ferrol A Coruña Spain

**Keywords:** Quality of Life, Students, Mental Health, Chronic Pain, Central Nervous System Sensitization, Calidad de Vida, Estudiantes, Salud Mental, Dolor Crónico, Sensibilización del Sistema Nervioso Central

## Abstract

**Background:**

University students are not exempt from physical and mental health problems. This study aimed to analyse the prevalence of musculoskeletal pain, central sensitization, health-related quality of life, and associated factors among health sciences students.

**Methods:**

Cross-sectional study conducted in Spanish health sciences students using anonymous, self-administered questionnaires. Data included sociodemographic characteristics of participants, health-related quality of life (SF-12), frequency and location of musculoskeletal pain (Nordic Musculoskeletal Questionnaire, Numeric Pain Rating Scale), and central sensitization (Central Sensitization Inventory, CSI). Factors associated with the SF-12 physical and mental component summaries (PCS and MCS) were identified using multiple lineal regression analysis.

**Results:**

Of the 338 participants, 76.3% were female. A high pre-valence of musculoskeletal pain was observed, particularly in the back region (e.g., 59% reported neck pain in the past month), with higher frequency in women. The mean PCS exceeded the Spanish adult population mean for both sexes (54.6 vs. 55.9; p = 0.02), whereas the mean MCS was lower than the population mean (36.7 vs. 42.8; p<0.001), even after comparing them with age- and sex-matched population standardized scores. Female sex, disability, chronic musculoskeletal pain, and CSI≥40 were associated with poorer physical health, while only CSI scores were associated with poorer mental health.

**Conclusions:**

Health sciences students show a high prevalence of musculoskeletal pain and significant deterioration in mental health. Central sensitization is strongly linked to worse outcomes. Women have poorer mental health and greater prevalence, severity, and chronification of musculoskeletal pain.

## RESUMEN

**Fundamento:**

Los estudiantes universitarios no están exentos de problemas de salud. El objetivo es analizar la prevalencia de dolor musculoesquelético, sensibilización central, calidad de vida relacionada con la salud y factores relacionados en estudiantes de ciencias de la salud.

**Metodología:**

Estudio transversal en estudiantes españoles de ciencias de la salud mediante cuestionarios anónimos y autoadministrados, incluyendo características sociodemográficas, calidad de vida relacionada con la salud (SF-12), frecuencia y localización de dolor musculoesquelético (Cuestionario Musculoesquelético Nórdico, Escala Numérica de Valoración del Dolor) y puntuación del Inventario de Sensibilización Central (CSI). Las variables relacionadas con el componente físico y mental (PCS y MCS) se determinaron mediante regresión lineal múltiple.

**Resultados:**

Respondieron 338 estudiantes, 76,3% mujeres. El dolor musculoesquelético fue muy prevalente, particularmente en la zona de la espalda (p.ej., 59% refirió dolor cervical en el último mes), más frecuente en las mujeres. La media de PCS fue superior a la de la población adulta española (54,6 vs. 55,9; p = 0,02), en ambos sexos, mientras que el MCS medio fue inferior a la media poblacional (36,7 vs. 42,8; p<0,001), incluso tras compararlo con las puntuaciones poblacionales estandarizadas de igual edad y sexo. Ser mujer, tener discapacidad, dolor musculoesquelético crónico y CSI≥40 se relacionaron con peor salud física, y la puntuación de CSI con peor salud mental.

**Conclusiones:**

Los estudiantes informaron alta prevalencia de dolor musculoesquelético y un deterioro significativo de su salud mental, con la sensibilización central asociada a peor salud. Las mujeres tienen peor salud mental y una mayor prevalencia, intensidad y cronificación del dolor musculoesquelético.

## INTRODUCTION

Musculoskeletal pain (MSP) is highly prevalent among university students^1^^-^^3^. It is a multifactorial condition that often becomes chronic, affects multiple regions, is more frequent in women, causes disability, and has substantial social and economic impact, particularly when involving back pain^4^^,^^5^. A strong bidirectional relationship between mental health and chronic MSP has also been reported^6^^,^^7^, especially among health sciences students^8^^-^^10^.

The central sensitisation hypothesis proposes that altered central pain processing contributes to the chronification and generalisation of MSP and its association with conditions such as widespread pain and depression^11^. However, clinical assessment of central sensitisation remains limited^12^. The Central Sensitisation Inventory, a self-report tool, helps identify symptoms and signs associated with this mechanism^12^.

Health-related quality of life (HRQoL) has increasingly become an important outcome, reflecting both its morbidity and healthcare resources use. Chronic MSP affects not only physical function but also mental well-being, making HRQoL assessment crucial^13^. The SF-36 and its shorter version SF-12 are widely validated instruments that evaluate physical and mental health and enables comparisons with reference population norms^6^^,^^14^^-^^16^.

Since the COVID-19 pandemic, the health of young people - particularly their mental health - has declined^17^. Health sciences students face similar challenges^18^^-^^21^, influenced by multiple academic and personal factors^8^^,^^9^^,^^20^^,^^21^. Their well-being is of particular concern given its potential impact of future professional performance and patient care.

Sex differences have been consistently linked to both mental health^8^^,^^9^^,^^19^ and MSP^2^^,^^3^^,^^5^. Although sex is considered non-modifiable, identifying populations at higher risk remains critical due to its interaction with modifiable factors^22^.

To date, no studies have examined the prevalence and characteristics of MSP, HRQoL and associated factors in Spanish health sciences students.

This study aims to (1) analyse the prevalence, intensity, and symptoms of chronic musculoskeletal pain in this population; (2) explore potential sex differences; (3) evaluate physical and mental HRQoL compared with sex- and age-standardised reference values; (4) identify factors associated with HRQoL.

## METHODS

An observational, descriptive, cross-sectional study was conducted among university students of legal age enrolled in a bachelor’s degree program in health sciences during the academic years 2020-21 or 2021-22 at a Spanish public university.

A total of 338 students participated. This sample size allowed estimation of the prevalence of MSP and central sensitisation with a margin of error of ±5.5% at a 95% confidence level, and estimation of mean HRQoL scores with a margin of error of ±1.1 points (assuming a standard deviation of 10 points).

The study was disseminated through social networks, posters, and faculty lectures between March and May of 2021 and 2022. Data were collected using an online form. After being informed of the objectives of the study, participants voluntarily and anonymously completed the data and questionnaires necessary for the research.

The study protocol was approved by the Research and Teaching Ethics Committee of the University of A Coruña (code 2021-004) and complied with current data protection regulations. The research was carried out in accordance with the principles of the Declaration of Helsinki.

The online form included socio-demographic questions and three validated questionnaires to assess MSP, pain intensity, central sensitisation, and HRQoL.

- *Socio-demographic variables*: age (years); gender (female, male, other); degree program (Nursing, Physiotherapy, Speech Therapy, Podiatry, Occupational Therapy); academic year (2020/21, 2021/22); sexual orientation (heterosexual, homosexual, bisexual, other); and disability status (yes/no, type: mental, visual, hearing, osteoarticular, other), given its potential relevance to health disparities and psychosocial well-being.
- *MSP prevalence and location*: assessed using the abbreviated Nordic Musculoskeletal Questionnaire^23^. Prevalence was reported as percentages by region and period.
- *Pain intensity*: measured with the Numerical Pain Scale, ranging from 0 (no pain) to 10 (worst pain imaginable).
- *Chronic MSP*: defined as the presence of pain in a given anatomical region across all assessed time periods.
- *Central sensitisation*: assessed using the *Central Sensitisation Inventory* (CSI)^24^. Part A includes 25 items on pain-related symptoms, psychosocial, cognitive, and functional aspects, scored from 0 (never) to 4 (always). The total score ranges from 0 to 100, with higher values indicating greater symptomatology. Severity scores are: subclinical (<29 points), mild (30-39), moderate (40-49), severe (50-59), and extreme (60-100). A score >40 was considered the cut-off for detection of symptoms. Part B records the presence (yes/no) of 10 diagnoses related to central sensitisation syndromes.
- *HRQoL*: evaluated using the SF-12v2 questionnaire, which measures physical and mental health during the previous four weeks. It consists of 12 items, answered on 3- or 5-point Likert scales, covering eight dimensions and providing two summary components: the Physical Component Summary (PCS) and the Mental Component Summary (MCS). Scores were first normalized to a 0-100 scale and subsequently converted into norm-based scores according to reference norms^16^. Values above or below 50 indicated better or worse health, respectively, compared with the Spanish reference population^16^^,^^25^. For each participant, the difference between their standardised score and sex- and age-matched reference norms was calculated and expressed as a z-score (standard deviations). These z-scores were multiplied by 10 for interpretability; a deviation exceeding 5 points (z>0.5 SD) was considered clinically meaningful^26^.

### Data Analysis

Descriptive analyses were performed using means and standard deviation (SD) for quantitative variables, and frequencies and percentages for qualitative variables, both overall and stratified by gender. Normality was assessed with the Kolmogorov-Smirnov test. Comparisons of quantitative variables were conducted using Student’s t test or the Mann-Whitney U test, depending on data distribution. Comparisons of categorical variables were performed using the Chi-square test or Fisher’s exact test when expected frequencies were small. Correlation between quantitative variables were examined using Spearman’s rho. Variables significantly associated with poorer HRQoL (based on PCS and MCS z-scores) were included in multivariate linear regression models. Associations were reported as regression coefficients (B) with 95% confidence intervals (95% CI). Two-tailed p-values <0.05 were considered statistically significant. Cases with missing data for a given variable were excluded from analyses involving that variable but retained for other analyses. All analyses were performed using SPSS v28.

## RESULTS

The majority of the 338 participants were female students (76.3%), with a similar mean age for both sexes (22.5 years; SD = 4.9). As no responses were recorded in the “other” category for gender, gender and sex were considered equivalent. Differences by sex were observed according to the degree program: more than half of the male students were enrolled in Physiotherapy (54%), while female students were more evenly distributed across Nursing, Physiotherapy, and Occupational therapy. Most responses were obtained from second-year students. Regarding sexual orientation, 76-79% of participants identified as heterosexual. Female students more frequently reported bisexuality, while homosexuality was more frequent among male students. Only 5% of the sample reported having some form of disability, more commonly visual and other types (Table 1).

**Table 1:** Sociodemographic characteristics, disability and sexual orientation for the total sample, and comparison by sex

	Global	Female	Male	p
	n=338	n=258 (76.3%)	n=80 (23.7%)	
Age (years), *mean (SD)*	22.5 (4.9)	22.4 (4.8)	22.6 (5.0)	0.790
Degree, *n (%)*				<0.001
Physiotherapy	112 (33.1)	69 (26.7)	43 (53.8)	
Nursing	100 (29.6)	85 (32.9)	15 (18.8)	
Occupational Therapy	72 (21.3)	58 (22.5)	14 (17.5)	
Podiatry	30 (8.4)	24 (9.3)	6 (7.5)	
Speech Therapy	24 (7.1)	22 (8.5)	2 (2.5)	
Course, *n (%)*				0.814
First	85 (25.1)	65 (25.2)	20 (25.0)	
Second	148 (43.8)	110 (42.6)	38 (47.5)	
Third	57 (16.9)	46 (17.8)	11 (13.8)	
Fourth	48 (14.2)	37 (14.3)	11 (13.8)	
Sexual Orientation, *n (%)*				-
Heterosexual	256 (76.6)	196 (76.0)	63 (78.8)	
Homosexual	12 (3.6)	6 (2.3)	6 (7.5)	
Bisexual	65 (19.2)	54 (20.9)	11 (13.8)	
Other	2 (0.6)	2 (0.8)	0 (0.0)	
Disability, *n (%)*				0.263
None	319 (94.7)	242 (93.8)	77 (97.5)	
Some disability	18 (5.3)	16 (6.2)	2 (2.5)	
Intellectual	1 (0.3)	1 (0.4)	0 (0.0)	
Visual	8 (2.4)	7 (2.7)	1 (1.3)	
Hearing	1 (0.3)	1 (0.4)	0 (0.0)	
Osteoarticular	2 (0.6)	2 (0.8)	0 (0.0)	
Other	6 (1.8)	5 (1.9)	1 (1.3)	

SD: standard deviation; p-value was obtained from Student’s t test for age and Chi-squared or Fisher exact test for qualitative variables; -: p-value could not be calculated due to low expected frequencies.

In terms of self-perceived health (SF-12), the mean MCS score was 38.2 (SD = 11.3), more than one SD below the reference mean of 50 in the general Spanish population. Female students had lower MCS scores compared with male students (36.7 vs. 42.8; p<0.001). For physical health, mean PCS scores were higher than population norms for both sexes (54.6 vs. 55.9; p = 0.021) (Table 2).

When each student´s score was compared with standardised values for individuals of the same age and sex, mental health scores were lower than the reference values. Among female students, the MCS was more than one standard deviation below the mean for Spanish women of the same age (-11.65). Among male students, the deficit was more than half a standard deviation (-8.68). By contrast, physical health scores were above reference values for both sexes (0.43 vs. 2.90, p<0.001). Overall, all individual domains yielded negative z-scores, indicating poorer self-perceived health compared with the reference population. Female students consistently presented lower z-scores than males across all domains, with significantly greater deviations from normative values. The largest negative differences were observed in the MCS components, particularly in Role Emotional, Vitality, Mental Health, and Social Function, highlighting a more pronounced decline in mental health indicators among women (Table 2).

**Table 2:** Health-related quality of life for the total sample of students, and comparison by sex (normalized scores and z-scores)

SF-12 Dimensions and summaries / Mean (SD)	Total (n=338)	Female (n=258)	Male (n=80)	p t-test
*Scores normalized with the Spanish population of the same sex and age group*				
Physical Function	52.4 (5.0)	51.9 (5.4)	53.8 (3.3)	<0.001
Role Physical	48.3 (8.0)	47.6 (8.2)	50.6 (6.7)	0.001
Bodily Pain	50.5 (6.5)	50.0 (6.9)	52.3 (4.5)	0.001
General Health	52.4 (7.1)	51.5 (7.7)	55.3 (6.3)	<0.001
Vitality	44.0 (7.6)	43.1 (7.6)	47.0 (6.9)	<0.001
Social Function	45.1 (9.8)	44.1 (10.2)	48.3 (7.6)	<0.001
Role Emotional	40.5 (11.6)	39.3 (11.7)	44.3 (10.5)	<0.001
Mental Health	42.7 (8.9)	41.4 (8.8)	47.0 (7.88)	<0.001
PCS	54.9 (5.8)	54.6 (6.2)	56.0 (4.3)	0.021
MCS	38.2 (11.3)	36.7 (11.3)	42.8 (10.3)	<0.001
*z-score, average deviation of the normalized score respect to the normalized score of the Spanish population of the same sex and age group*				
Physical Function	-0.86 (4.99)	-1.33 (5.35)	0.66 (3.18)	<0.001
Role Physical	-4.04 (7.99)	-4.88 (8.17)	-1.34 (6.77)	<0.001
Bodily Pain	-0.93 (6.57)	-1.71 (6.89)	1.58 (4.63)	<0.001
General Health	-1.50 (7.05)	-2.03 (7.19)	0.23 (6.29)	0.012
Vitality	-7.57 (7.54)	-8.00 (7.65)	- 6.15 (7.00)	0.054
Social Function	-6.17 (9.75)	-7.02 (10.19)	-3.44 (7.65)	0.001
Role Emotional	-9.71 (11.50)	-10.46 (11.70)	-7.30 (10.50)	0.024
Mental Health	-7.11 (8.70)	-7.83 (8.84)	-4.76 (7.83)	0.006
PCS	1.01 (5.80)	0.43 (6.10)	2.90 (4.19)	<0.001
MCS	-10.95 (11.11)	-11.65 (11.27)	-8.68 (10.32)	0.036

SD: standard deviation; **: PCS: Physical Component Summary; MCS: Mental Component Summary.

Regarding the prevalence of MSP in the previous year, month, week, and on the day of assessment (by anatomical site and sex), back regions (neck, upper back, and lumbar) were the most commonly affected across all time frames. For example, in the cervical region, MSP was reported by 69.4% of females and 62.5% of males in the past 12 months (p = 0.250). Although slightly lower for shorter recall periods, prevalence remained high: 59.3% vs 51.2% in the past month (p = 0.203), 50.4% vs 43.8% in the past week (p = 0.299), and 34.5 % vs 23.8% on the day of the survey (p = 0.072) for females and males, respectively. Chronic neck pain was reported by 32.2% of females and 20.0% of males (p = 0.037). Female students showed a higher prevalence of MSP across nearly all regions and time frames. Moreover, a higher percentage of female than male students reported MSP in three or more regions. For chronic pain, at least 25% of female students reported persistent pain in any back region, whereas about 20% of male students reported chronic pain in the neck and lumbar regions (Table 3).

**Table 3:** Prevalence (%) of musculoskeletal pain for the ten anatomical regions surveyed using the Nordic Musculoskeletal Questionnaire by frequency of pain and by sex

	MSP in the last									MSP today			Chronic pain		
	12 months			month			week								
	F	M	p	F	M	p	F	M	p	F	M	p	F	M	p
*Locations with MSP*															
Neck	69.4	62.5	*0.250*	59.3	51.2	*0.203*	50.4	43.8	*0.299*	34.5	23.8	*0.072*	32.2	20.0	*0.037*
Upper back	64.3	47.5	*0.007*	53.9	35.0	*0.003*	44.6	26.3	*0.004*	27.9	12.5	*0.005*	27.1	12.5	*0.007*
Lumbar	64.7	53.8	*0.077*	55.8	41.3	*0.023*	44.6	36.3	*0.188*	27.1	20.0	*0.201*	25.6	18.8	*0.211*
Shoulder-arm	36.0	37.5	*0.813*	25.6	26.3	*0.905*	20.2	22.5	*0.651*	12.4	10.0	*0.561*	11.2	10.0	*0.756*
Elbow-forearm	7.8	7.5	*0.941*	5.8	3.8	*0.580*	4.3	2.5	*0.474*	0.8	0.0	*0.999*	0.8	0.0	*0.999*
Wrist-hand	23.3	17.5	*0.277*	17.1	7.5	*0.035*	11.2	7.5	*0.337*	5.4	5.0	*0.999*	4.3	1.3	*0.307*
Abdomen	31.0	6.3	*<0.001*	23.6	7.5	*0.002*	19.4	5.0	*0.002*	7.8	2.5	*0.096*	4.7	2.5	*0.533*
Hip-thigh	19.4	23.8	*0.397*	11.2	13.8	*0.544*	9.7	11.3	*0.685*	5.8	3.8	*0.580*	5.4	3.8	*0.771*
Knee-calf	31.8	30.0	*0.764*	23.6	21.3	*0.657*	20.9	15.0	*0.242*	10.9	7.5	*0.384*	9.3	5.0	*0.223*
Ankle-foot	26.7	25.0	*0.757*	22.5	17.5	*0.342*	18.2	15.0	*0.508*	7.0	12.5	*0.117*	5.4	12.5	*0.031*
*Number of locations with MSP*															
*Median (IQR)*	*4.0 (2-5)*	*3.0 (2-4)*		*3.0 (2-4)*	*2.0 (1-3)*		*2.0 (1-4)*	*2.0 (1-3)*		*1.0 (0-2)*	*1.0 (0-2)*		*1.0 (0-2)*	*1.0 (0-1)*	0.040*
None	7.0	15.0	0.030	8.9	16.3	0.005	16.3	21.3	0.020	36.0	46.3	0.004	39.1	48.8	0.230
One	8.5	6.3		15.9	18.8		21.3	21.3		26.7	25.0		26.7	27.5	
Two	15.2	25.0		20.2	25.0		16.7	28.7		15.9	18.8		15.1	15.0	
Three	17.8	7.5		19.0	17.5		18.6	16.3		10.5	6.3		10.5	6.3	
More than three	51.6	46.4		36.1	22.6		27.1	12.7		10.9	3.8		8.5	2.5	

MSP: musculoskeletal pain; F: female; M: male. Chronic pain: the presence of MSP in all the periods studied for each of the ten locations. *: p-value obtained by the Mann-Whitney U test; all proportions were compared by Chi-square test or Fisher’s exact test.

According to the Pain Numerical Rating Scale scores, the most painful regions in the previous week were lumbar, cervical, and upper back, as well as the hips. Pain intensity was significantly greater among female students in the neck, upper back, and lumbar regions. For the wrist-hand, abdomen, hip-thigh, and ankle foot, mean pain intensity was higher in male students, although these differences were not statistically significant (Table 4).

**Table 4:** Intensity of musculoskeletal pain (median and range) measured in ten anatomical locations in the last week

Body location	Global	Female	Male	p*
Neck	5 (1-10)	5 (4-6)	4 (3-56)	0.011
Upper back	5 (3-6)	5 (3-6)	4 (2-5)	0.001
Lumbar	5 (1-10)	5 (4-7)	4 (2-6)	0.021
Shoulder-Arm	4 (3-6)	5 (3-6)	4 (3-6)	0.232
Elbow-Forearm	3 (2-6,5)	3 (2-3)	3 (2-6)	0.575
Wrist-Hand	4 (2,5-5)	4 (3-7)	4 (3-5)	0.799
Abdomen	4 (3-6)	4 (3-6)	5.5 (1.5-7,5)	0.716
Hip-Thigh	5 (3-7)	5 (2,5-6,5)	5 (3-7)	0.752
Knee-Calf	5 (3-6)	5 (3-6)	5 (3-6)	0.627
Ankle-Foot	4 (3-6)	4 (3-6)	4.5 (3-6.5)	0.491

SD: standard deviation; *: Mann-Whitney U test p-values.

**Table 5:** Central Sensitization Inventory scores for the total sample and by sex

	Total (n=338)	Female (n=258)	Male (n=80)	p
**Overall score of the Central Sensitization Inventory, *mean (SD)***	29.6 (13.3)	32.0 (13.1)	21.8 (10.9)	<0.001*
Categorized score, *n (%)*				<0.001
Subclinical (≤29)	183 (54.1)	121 (46.9)	6 (77.5)	
Average (30-39)	84 (24.9)	71 (27.5)	13 (16.3)	
Moderate (40-49)	42 (12.4)	40 (15.5)	2 (2.5)	
Severe (50-59)	20 (5.9)	17 (6.6)	3 (3.8)	
Extreme (60-100)	9 (2.7)	9 (3.5)	0	
Part B, *n (%)*				
Restless legs syndrome	0	0	0	-
Chronic fatigue syndrome	1 (0.3)	1 (0.4)	0	0.999
Fibromyalgia	1 (0.3)	1 (0.4)	0	0.999
Temporomandibular disorders	23 (6.8)	19 (7.4)	4 (5.0)	0.463
Migraine/Tension headaches	54 (16.0)	47 (18.2)	7 (8.8)	0.043
Irritable bowel syndrome	13 (3.8)	12 (4.7)	1 (1.3)	0.315
Multiple chemical sensitivity	1 (0.3)	1 (0.4)	0	0.999
Whiplash	23 (6.8)	12 (4.7)	11 (13.8)	0.005
Anxiety or panic attacks	66 (19.5)	62 (24.0)	4 (5.0)	<0.001
Depression	37 (10.9)	32 (12.4)	5 (6.3)	0.124

SD: standard deviation; *: Student’s t test p-value; all proportions were compared by Chi-square test or Fisher’s exact test; Anxiety proportions calculated over n=258.

On the CSI, mean scores were 29.6 (SD = 13.3), which is within the *average* range; 79% of parti-cipants scored below 40 (Table 5). Female students scored higher than male students did (32.0 vs. 21.8; p<0.001), with a greater proportion presenting moderate or extreme central sensitization (25.6 vs. 6.3%).

**Figure 1:**
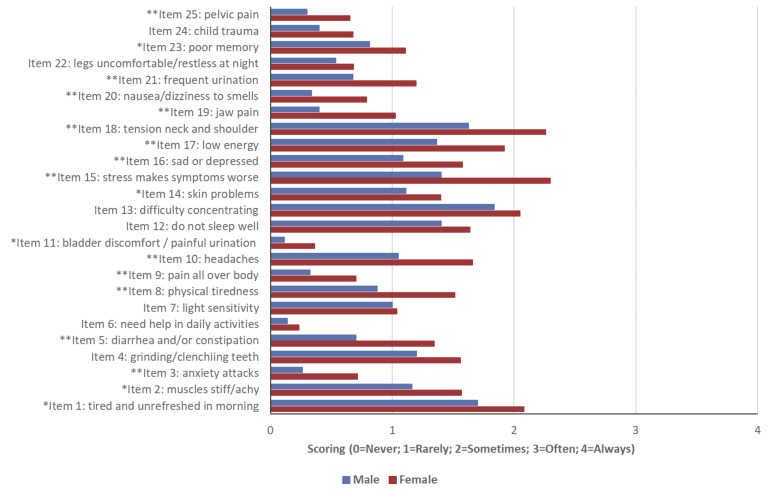
Distribution of Central Sensitization Inventory scores for each item, according to sex.

Figure 1 shows the distribution of scores for each CSI item, stratified by sex. Women reported higher scores across all items, indicating a greater frequency and intensity of central sensitization-related symptoms. The most affected items in both sexes included: item 15 (“stress makes symptoms worse”), item 18 (“tension in neck and shoulders), item 1 (“tired and unrefreshed in the morning”), and item 13 (“difficulty concentrating”). The largest sex difference was observed in item 15, with female students reporting significantly higher scores than males.

*p<0.05; ** p=<0.001. **Item 1**: I feel tired and discouraged when I get up in the morning. **Item 2:** My muscles are tense and sore. **Item 3**: I have panic attacks. **Item 4**: I grind my teeth or clench my jaw. **Item 5**: I have problems with diarrhea or constipation. **Item 6**: I need help to carry out my daily activities. **Item 7**: I am sensitive to bright light. **Item 8:** I get tired easily when I am physically active. **Item 9**: I feel pain all over my body. **Item 10:** I have headaches. **Item 11**: I have discomfort in my bladder or a burning sensation when I urinate. **Item 12:** I do not sleep well. **Item 13**: I have difficulty concentrating. **Item 14**: I have skin problems such as dryness. itching or rashes. **Item 15:** Stress makes my physical symptoms worse. **Item 16**: I feel sad or depressed. **Item 17:** I feel low in energy. **Item 18**: I have muscle tension in my neck and shoulders. **Item 19:** I have pain in my jaw. **Item 20**: Some smells. such as perfumes. make me feel dizzy and nauseous. **Item 21:** I have to urinate frequently. **Item 22:** My legs feel uncomfortable and restless when I try to sleep at night. **Item 23:** I have difficulty remembering things. **Item 24:** I suffered some trauma as a child. **Item 25:** I have pain in my pelvic area.

Regarding the presence of comorbid syndromes (part B of the CSI), female students most frequently reported anxiety or panic attacks, migraine or tension-type headaches and depression, while a history of cervical sprain was more common among male students.

Age was not correlated with PCS (rho = -0.06; p = 0.277) or MCS (rho = -0.07; p = 0.228). In contrast, CSI scores showed negative correlations with both component summaries (p<0.001): rho = -0.26 for PCS and rho = -0.64 for MCS.

Factors associated with PCS and MCS are presented in Table 6. In bivariate analysis, worse physical health was associated with female sex, presence of any disability, chronic MSP at any site, and a CSI score ≥40; all four variables remained significant in the multivariate model. For mental health (MCS), after adjusting for sex, sexual orientation, and chronic MSP, significant associations were observed with chronic MSP at any site and with sexual orientation other than heterosexual. After adjustment for CSI scores, the CSI remained the only variable significantly associated with worse mental health.

**Table 6:** Variables related to physical and mental component summaries from SF-12 z-scores

	Physical Component Summary				Mental Component Summary			
	Univariate analysis		Multivariate analysis		Univariate analysis		Multivariate analysis	
	Mean (SD)	p	B (95%CI)	p	Mean (SD)	p	B (95%CI)	p
*Sex*		*<0.001*		*0.035*		*0.036*		*0.732*
Male	2.9 (4.2)		1		-8.7 (10.3)		1	
Female	0.4 (6.1)		-1.5 (-2.9; -0.1)		-11.7 (11.3)		-0.5 (-3.0; 2.1)	
*Disability*		*0.023*		*<0.001*		*0.156*		
No	1.3 (5.3)		1		-10.8 (11.1)			
Any	-4.6 (10.4)		-4.6 (-7.2; -2.0)		-14.1 (11.3)			
*Sexual orientation*		*0.798*				*0.005*		*0.071*
Heterosexual	1.1 (5.7)				-10.0 (11.0)		1	
Homo-/bisexual/other	0.9 (6.2)				-14.0 (10.9)		-2.3 (-4.9; 0.2)	
*Chronic pain in any location*		*<0.001*		*0.001*		*0.014*		*0.800*
No	2.8 (4.7)		1		-9.2 (11.0)		1	
Yes	-0.2 (6.2)		-2.1 (-3.3; -0.9)		-12.2 (11.0)		-0.2 (-2.2; 1.9)	
*1 location*	1.2 (5.5)				-12.4 (11.7)			
*2 locations*	1.1 (4.9)				-12.7 (9.5)			
*3 locations*	-2.6 (6.2)				-9.8 (11.5)			
*>3 locations*	-5.3 (7.4)				-13.8 (10.9)			
*Central Sensitization*		*<0.001*		*<0.001*		*<0.001*		*<0.001*
No symptoms	1.9 (4.8)				-8.5 (10.0)		1	
Symptoms	-2.8 (7.7)		-3.2 (-4.8; -1.6)		-21.3 (9.6)		-12.3 (-15.2; -9.3)	

CI: Confidence interval; SD: Standard deviation; univariate p-values were obtained from Student’s t test

## DISCUSSION

This study characterises undergraduate health science students in terms of the prevalence, location, generalisation, chronicity, and intensity of MSP. It also analyses their self-perceived physical and mental HRQoL and compares it with standardised values for the Spanish population. In addition, differences between genders are examined.

Three main findings emerge. First, these young people show a high prevalence of MSP, particularly in the back. The pain is often generalised, chronic, of moderate intensity, and differs by sex. Second, the study shows that these students report slightly better physical health and significantly worse mental health than the Spanish population of the same sex and age. Third, poorer physical health is associated with being female, having a disability, chronic MSP, and symptoms of central sensitization, while poorer mental health is associated with central sensitization symptoms.

The high prevalence of MSP among university students observed in this study is consistent with previous research. However, this study provides a more detailed characterization of pain. The back is identified as the most common pain site, in agreement with other studies^1^^-^^3^^,^^10^. It is noteworthy that MSP is reported in multiple locations, often in a chronic form, an aspect that has been less frequently considered in previous studies^10^.

With respect to gender differences in MSP, female students report a higher prevalence of upper back pain, greater intensity of back pain, and more pain-affected sites, consistent with prior studies^1^^,^^2^^,^^5^. Furthermore, CSI results suggest a higher prevalence of physical symptoms related to pain among female students. Despite being young and generally functional, many students exhibit medium-level sensitization symptoms (according to CSI classification). This finding points to the need for preventive measures in this population, given the recent recognition of the predictive value of CSI scores^11^ and pain-related disability^27^.

Although casualty cannot be established, the academic environment of health science students may contribute to the high prevalence of MSP. This may be related to exposure to physical risk factors (e.g., lifting during clinical training, repetitive postures, or demanding movements^28^, as well as study-related stressors^7^^,^^10^.

It is unsurprising that PCS scores in this group are slightly higher than in the general population of the same age. The variables associated with poorer PCS are consistent with previous studies^6^^,^^16^^,^^29^^,^^30^.

Of particular concern, however, is the significant deterioration in mental health, with scores falling well below reference scores. For female students, the difference exceeds one standard deviation, and for male students it approaches one standard deviation. The findings of the CSI support these results, with over 10% of students diagnosed with depression and 20% diagnosed with anxiety.

Although no similar studies have been found among Spanish university students using the SF-12 to compare these results, reference values are available^16^. However, the population values were obtained in a different social and health context (prior to the COVID-19 pandemic) and do not differentiate between university students and other young people, but, results align with the barometer conducted among adults aged 15-29 years, indicating a decline in their mental health^31^.

Additionally, studies of university students in other countries have reported similar SF-12 scores^9^^,^^20^. In a study with around 4,000 students, the MCS score was more than one standard deviation (-11.8) below normalized values^20^, in line with our findings. Other research, using measures different from SF-12, have also identified moderate levels of mental health distress among health sciences students^8^^,^^18^^,^^19^^,^^21^. Regarding the variables associated with worse mental health, only symptoms of central sensitisation were identified. While studies on this kind of symptoms in this population are scarce, previous research in patients with chronic pain has demonstrated a correlation between central sensitisation symptoms and the SF-36 MCS^30^^,^^32^. It is plausible that physical symptoms contribute to poorer mental health^33^, although it cannot be established. This hypothesis is reinforced by our analyses: in models without CSI scores, chronic MSP negatively affects mental health. In line with previous studies, being female^8^^,^^16^ and having a non-heterosexual orientation^34^ are also associated with poorer mental health.

Several limitations should be considered when interpreting the results of this study. First, selection bias is possible, as students more concerned about MSP may have been more willing to participate. To minimise this risk and maximise participation, data collection was conducted during a compulsory, face-to-face class. Nevertheless, the sample size remains limited. Second, all data were self-reported. Although participants were assured of anonymity and instructed to respond accurately, self-reporting may introduce bias. However, SF-12 has shown good concordance with clinical outcomes^14^. Third, while observed gender differences are consistent with other studies, they may be spurious, as gender-specific analysis were not pre-specified. Moreover, female students are overrepresented in health science programmes, limiting generalisability to other university populations.^20^. Finally, the cross-sectional design precludes establishing causal relationships and findings from inferential analyses, and should therefore be interpreted with caution.

In conclusion, undergraduate health science students report high prevalence of MSP, particularly in the back, often generalised, chronic, and of moderate intensity. They also show marked deterioration in mental health, despite physical HRQoL being comparable to that of the general population. Medium-level central sensitisation symptoms are observed and are associated with poorer mental health. Gender differences are evident: women report worse mental health, greater intensity and frequency of back pain, more affected sites, and more sensitisation symptoms.

The relevance of these findings lie in the high prevalence of MSP and the deterioration of mental health in this population group. Academic and health managers should consider these issues when designing preventive measures. Addressing the needs of the most vulnerable students should be a priority, given the potential impact on their future health, professional careers, and patient care. Evidence-based strategies to reduce psychological^35^^)^ and physical^36^ symptoms exist, and their implementation may be particularly beneficial for this group.

## Data Availability

The data that support the findings of this study are openly available in Zenodo at https://doi.org/10.5281/zenodo.7782495
